# Supplementary material for: Resolving the full spectrum of human genome variation using Linked-Reads
Source: Genome Res. 2019 Apr;29(4):635–45. doi: 10.1101/gr.234443.118 (PMC6442396; doi:10.1101/gr.234443.118)
Supplement: Supplemental Material [file supp_gr.234443.118_Supplemental_longranger-2.2.zip › longranger-2.2.tar.bz2!/lr2.2-public/lib/stan/cmdstan/stan/doc/api/html/csv_8hpp_source.html]

Stan: src/stan/common/recorder/csv.hpp Source File


|  |  |
| --- | --- |
|  | Stan  2.5.0  probability, sampling & optimization |


- Main Page
- Related Pages
- Namespaces
- Classes
- Files

- File List
- File Members

All Classes Namespaces Files Functions Variables Typedefs Enumerations Enumerator Friends Macros Pages

- src
- stan
- common
- recorder

csv.hpp

Go to the documentation of this file.

1 #ifndef STAN\_\_COMMON\_\_RECORDER\_\_CSV\_HPP

2 #define STAN\_\_COMMON\_\_RECORDER\_\_CSV\_HPP

3

4 #include <ostream>

5 #include <string>

6 #include <vector>

7

8 #include <stan/math/meta/index\_type.hpp>

9

10 namespace stan {

11

12  namespace common {

13

14  namespace recorder {

15

19  class csv {

20  private:

21  std::ostream \*o\_;

22  const bool has\_stream\_;

23  const std::string prefix\_;

24

25  public:

31  csv(std::ostream \*o, std::string prefix)

32  : o\_(o), has\_stream\_(o != 0), prefix\_(prefix) { }

33

44  template <class T>

45  void operator()(const std::vector<T>& x) {

46  typedef typename stan::math::index\_type<std::vector<T> >::type idx\_t;

47  if (!has\_stream\_)

48  return;

49

50  if (x.size() != 0) {

51  \*o\_ << x[0];

52  for (idx\_t n = 1; n < x.size(); n++) {

53  \*o\_ << "," << x[n];

54  }

55  }

56  \*o\_ << std::endl;

57  }

58

68  void operator()(const std::string x) {

69  if (!has\_stream\_)

70  return;

71  \*o\_ << prefix\_ << x << std::endl;

72  }

73

78  void operator()() {

79  if (!has\_stream\_)

80  return;

81  \*o\_ << std::endl;

82  }

83

89  bool is\_recording() const {

90  return has\_stream\_;

91  }

92  };

93

94

95  }

96  }

97 }

98

99 #endif

stan::common::recorder::csv::operator()

void operator()(const std::string x)

Print single string with a prefix.

**Definition:** csv.hpp:68

stan::common::recorder::csv::operator()

void operator()(const std::vector< T > &x)

Print vector as csv.

**Definition:** csv.hpp:45

stan::common::recorder::csv

Writes out a vector as string.

**Definition:** csv.hpp:19

stan::common::recorder::csv::is\_recording

bool is\_recording() const

Indicator function for whether the instance is recording.

**Definition:** csv.hpp:89

stan::math::index\_type

Primary template class for the metaprogram to compute the index type of a container.

**Definition:** index\_type.hpp:21

stan::common::recorder::csv::csv

csv(std::ostream \*o, std::string prefix)

Construct an object.

**Definition:** csv.hpp:31

index\_type.hpp

stan::common::recorder::csv::operator()

void operator()()

Prints a blank line.

**Definition:** csv.hpp:78


---

[ Stan Home Page ]

*© 2011–2014,
Stan Development Team.*
